# Raman Spectroscopic Detection of Anthrax Endospores in Powder Samples**

**DOI:** 10.1002/anie.201201266

**Published:** 2012-04-13

**Authors:** S Stöckel, S Meisel, M Elschner, P Rösch, J Popp

**Affiliations:** Institut für Physikalische Chemie, Friedrich-Schiller-Universität JenaHelmholtzweg 4, 07743 Jena (Germany); Institut für Photonische TechnologienAlbert-Einstein-Strasse 9, 07745 Jena (Germany); Friedrich Loeffler Institut, Bundesforschungsinstitut für Tiergesundheit, Institut für bakterielle Infektionen und ZoonosenNaumburger Strasse 96a, 07743 Jena (Germany)

**Keywords:** analytical methods, anthrax, endospores, Raman spectroscopy

Polymerase chain reaction (PCR) is becoming the method of choice for detecting microorganisms concealed in complex matrices or “hoax materials” like household dry powders, food, and soil.[1] However, adding samples directly to a PCR reaction is in most cases not possible because of the presence of PCR inhibitors in the sample.[2] Thus, in order to reliably use PCR, one must either enrich the culture prior to the analysis, which is time-consuming for fastidious organisms, or extract the total DNA directly from the sample, which requires an extraction technique capable of processing different sample types. However, since most DNA extraction methods are often not generic in that sense, they lead to unreliable DNA recovery yields.[2, 3]

By combining microscopy and Raman spectroscopy with visible-light excitation, it is possible to probe bacteria at the single-cell level making biomass-enrichment steps prior to analysis unnecessary.[4] The whole organism is characterized by its Raman spectrum comprising information about the intracellular, membrane, and surface material of the cells. The Raman spectral fingerprints of the bacteria can be compared to reference spectra of the same or related species such that bacteria can be identified in various possible civilian and military scenarios, for example, *Bacillus anthracis*, the etiological agent of the acute disease anthrax.[5] Several publications have dealt with testing for *Bacillus* endospores embedded in hoax materials and mail letters, but these relied solely on detecting the endospore-specific substance calcium dipicolinate (CaDPA).[6] This strategy is limited, however, since nonpathogenic bacilli other than *B. anthracis* may deliver false alarms.

We report here the first application of Raman spectroscopy to detect and identify anthrax endospores in environmental samples, even in the presence of other *Bacillus* species. Our suggested process provides results within 3 h after sample removal with minimal investment of material and time: First, the contaminated samples (roughly 100 mg) must be inactivated for 1 h with formaldehyde solution to kill possible pathogens.[7] The subsequent endospore extraction based on density-gradient centrifugation takes up to 30 min, before a microliter of the final suspension is dried on fused-silica plates and probed with a micro-Raman setup (6 s per endospore, 532 nm excitation).[8] Finally the obtained endospore Raman spectra are compared by means of chemometric analysis and a spectral database.

We focused particularly on powders, because they are among the most common nonclinical types of samples to be tested for *B. anthracis*.[9] To cover a broad range of sample types we selected seven household powders (baking powder, gypsum, milk powder, baking soda, analgesic tablet, bird sand, washing detergent) and spiked them with endospores of two strains of *B. anthracis* plus four other *Bacillus* species. The genetically closely related *B. anthracis*, *B. mycoides*, and *B. thuringiensis* belong to the *Bacillus cereus* clade, which often provoke cross-reactions with each other in PCR assays.[10] More distant species are the soil saprophytes *B. megaterium* and *B. subtilis*.

Studies performed on cynomolgus monkeys led to the assumption that the LD_50_ value for humans is in the range of around 8000 to 50 000 colony forming units (cfu) for aerosolized anthrax spores, which is equivalent to roughly 8–50 ng.[11] We inoculated defined spore loads into baking powder and bird sand samples to test whether the chosen isolation procedure is sensitive enough. Both matrices were spiked with viable *B. thuringiensis* endospores in concentrations of 10^8^, 10^6^, 10^4^, and 10^3^ cfu per gram of matrix. The recovered endospores were enumerated by viable cell counting (Table S1 in the Supporting Information): 1 % to 12 % of the initial cells were isolated, which is sufficient, since 100 endospores per sample are enough to give reasonable results in the following Raman measurements. A 1 μL portion of each of the prepared samples was analyzed by Raman spectroscopy on the single-particle level as illustrated in Figure 1 for a processed baking powder sample spiked with *B. anthracis* Sterne. A dark-field image is transformed into a binary image to assess particles according to morphological features. The five labeled particles in Figure 1 b were then measured; their unprocessed Raman spectra are shown in Figure 1 c (i–iv: endospores, v: poly(3-hydroxybutyrate), a common bacterial metabolite). In this way, roughly 50 particles per sample were measured with an integration time of 5 s plus 1 s preburning time to mitigate the spectral contributions of fluorescence, though this was already a minor factor in most of the endospore spectra.

**Figure 1:**
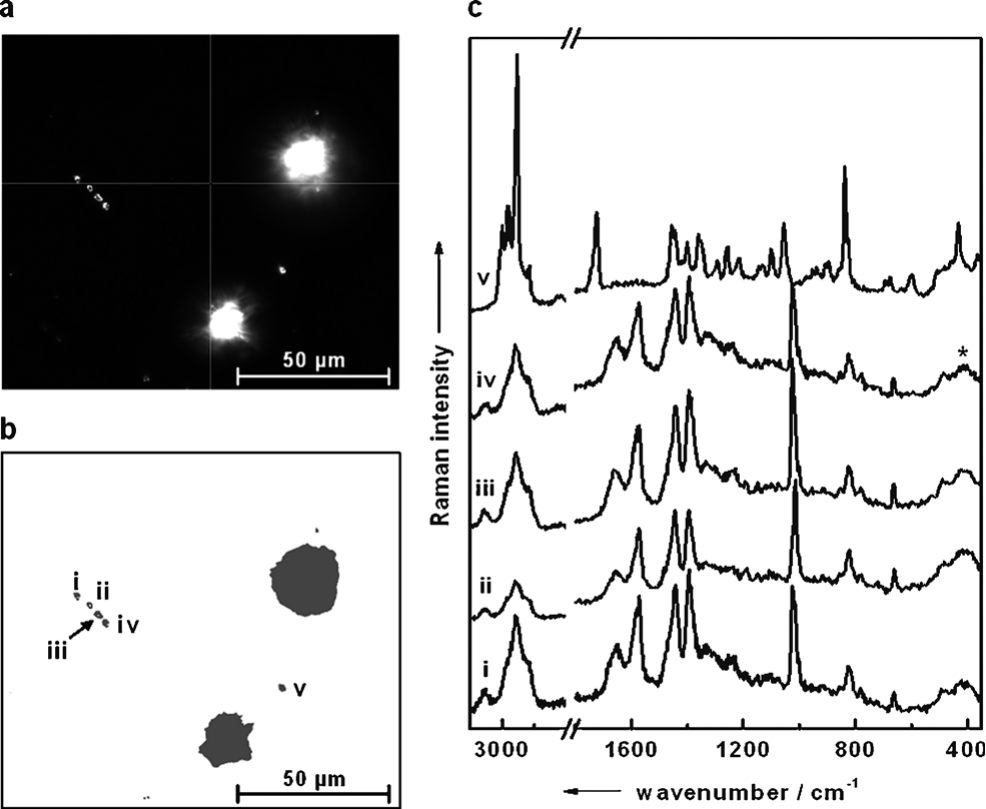
Particle analysis applied on a baking powder sample spiked with *B. anthracis* Sterne endospores. a) Dark-field-illuminated field of view. b) Five particles (i–v) match the morphological criteria for bacterial cells in the binary image. c) Unprocessed Raman spectra of the particles i–v. The asterisk denotes a band from the fused-silica substrate.

Figure 2 a displays mean Raman spectra of each of the analyzed *Bacillus* species. Dominating features in the spectra are mainly bands arising from the endospore-specific salt CaDPA at 657, 1013, and 1397 cm^−1^. Other spectral contributions arise exclusively from proteins, for example bands at 1001 cm^−1^ (ring-breathing vibration of phenylalanine) and 1659 cm^−1^ (amide I), and are complemented by signals from nucleic acids like the band at 781 cm^−1^ (ring vibration of cytosine/uracil). Some bands can be assigned to superpositions of signals from different biomolecules with CaDPA, for example the bands at 821 cm^−1^ (superposition of the ring-breathing mode of tyrosine with the CaDPA carboxylate stretching mode), at 1450 cm^−1^ (the CH_2_/CH_3_ deformation mode of proteins and lipids), and at 1578 cm^−1^ (ring vibration of guanine and adenine with pyridine ring vibrations of CaDPA). The intense signal in the high-wavenumber region at 2939 cm^−1^ is due to symmetric and asymmetric CH stretching vibrations of mainly proteins and lipids.[12]

**Figure 2:**
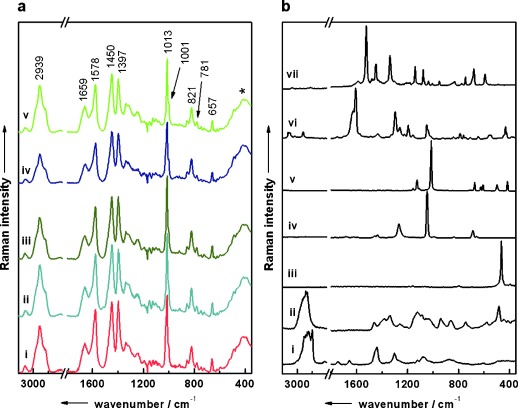
Raman spectra of *Bacillus* endospores and various matrix particles. a) Background-corrected mean Raman spectra of *B. anthracis* (i, calculated from 997 single-endospore Raman spectra), *B. megaterium* (ii, 1420 spectra), *B. mycoides* (iii, 1142 spectra), *B. subtilis* (iv, 1217 spectra), and *B. thuringiensis* (v, 947 spectra). The asterisk denotes a band from the fused-silica substrate. b) Raman spectra of particles of powdered milk (i, milk fat), baking powder (ii, starch), bird sand (iii, quartz), baking soda (iv, sodium bicarbonate), gypsum (v, calcium sulfate dihydrate), analgesic tablet (vi, acetylsalicylic acid), and washing powder (vii, copper phthalocyanine). The spectra are scaled and offset vertically for clarity.

In samples with low bacterial load, not only endospores but also biotic and abiotic matrix particles with endospore-like appearance might be probed in this way. Spectra of these clutter materials may disturb the subsequent statistical evaluation, since the spectral libraries cannot contain spectra of all possible matrix material. Figure 2 b displays a collection of Raman spectra of matrix particles encountered during the measurements. An array of strongly distinctive Raman spectra is visible and stands in stark contrast to the Raman spectra of endospores (Figure 2 a). It is therefore not a problem if matrix particles are measured, since their spectra can easily be recognized and sorted out.

A proper data evaluation step is the third mainstay for our concept. We selected a linear discriminant analysis (LDA) as the algorithm of choice. This classifier was recently applied to discriminate bacteria and particles of inorganic origin according to their Raman spectra.[13] To perform the classification problem at hand the algorithm was trained to distinguish between Raman spectra of the five different species. Thus, a collection of Raman spectra of known origin was fed into the algorithm to define discriminant functions for the best discrimination between the groups. Endospores from at least ten independently cultivated batches for each nonpathogenic *Bacillus* species were either taken directly from the culture medium or were inoculated into the powder matrices for at least 24 h, inactivated, isolated, and analyzed by Raman spectroscopy. This was done twice for every endospore–matrix combination (for *B. anthracis* only baking powder and bird sand) leading to a model database with a total of 5723 Raman spectra (Table S2 a in the Supporting Information). The considerable partitioning of the data due to three of four discriminant functions is shown in Figure S1 in the Supporting Information. After the discrimination functions were parameterized a cross-validation was performed: 5427 Raman spectra were labeled correctly (94.8 %, Table S3 in the Supporting Information). Most of the false negatives occurred between *B. mycoides* and *B. thuringiensis*; most of the wrongly labeled *B. anthracis* spectra could also be found in these two classes. Apparently the spectra of the *Bacillus cereus* class are more similar than those of the other classes.

To simulate the analysis of unknown real-world samples, samples spiked with new batches of the five *Bacillus* species were prepared. In this way we could also estimate the model’s propensity to overfit the data. If the model is too specific to the samples in the training data set, the model generalization potential would be drastically lowered. This new set of 1650 spectra with “unknown identities” (Table S2 b in the Supporting Information) was tested against the aforementioned LDA model. In Figure 3 a the data have been rearranged after the spectra had been projected on two of the LD axes. The clustering of the data is pronounced; that is, data for each single class are pooled together in coherent point clouds, which are mainly located within the classification model’s confidence intervals (double standard deviation, depicted as ellipses) of the respective class. The confusion table (Table 1) reflects the identification accuracies for each species, giving an overall accuracy of 96.8 %. The highest and lowest rates are reported for *B. subtilis* (100 %) and *B. thuringiensis* (91.2 %), respectively; specifically for *B. anthracis* a sensitivity of 99.6 % was achieved. Falsely labeled spectra occurring for *B. anthracis*, *B. mycoides*, and *B. thuringiensis* were assigned to one of these three classes. All in all, the recognition rates for the new set of spectra are in the range of those achieved by cross-validation by using model inherent spectra. This can be said for all the different sample types, since no matrix type included in the model obviously corrupted the endospore spectra.

**Figure 3:**
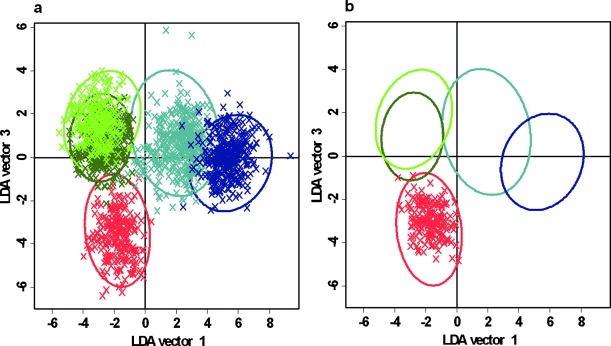
LDA score plots of the validation data. The ellipsoids display the model confidence intervals (double standard deviation) for each class. a) *B. anthracis* (red), *B. megaterium* (cyan), *B. mycoides* (olive-green), *B. subtilis* (blue), and *B. thuringiensis* (light green). b) The red crosses represent 191 spectra of *B. anthracis* 367 endospores, which were isolated from a matrix (table salt) unknown to the LDA model.

**Table 1:** Results of the validation experiment with an independent dataset[a]

Identified as[b]	Bant	Bmeg	Bmyc	Bsub	Bthu	Bant [C]
Bant	**241**	4	3	0	0	**183**
Bmeg	0	**374**	0	0	0	0
Bmyc	0	0	**382**	0	26	8
Bsub	0	0	0	**331**	0	0
Bthu	1	2	17	0	**269**	0
Sens. [%]	99.6	98.4	95.0	100	91.2	95.8

[a] The species-wise sensitivities (sens.) are given. The overall rate was 96.8 %. The last column reflects the labeling of endospores isolated from table salt [C].

[b] Bant=*B. anthracis*, Bmeg=*B. megaterium*, Bmyc=*B. mycoides*, Bsub=*B. subtilis*, Bthu=*B. thuringiensis*.

To test whether a sample type yet unknown to the model can be handled, we spiked common table salt with *B. anthracis* and processed it in the described way. 191 Raman spectra of isolated spores were measured and labeled by using the model. In Figure 3 b most of them (183/191 spectra, 95.8 %, Table 1) were put into the area populated by the *B. anthracis* data of the model and misclassified spectra were solely labeled to be members of the *B. mycoides* class. However, it is apparent that satisfactory identification rates can be achieved even when the endospores originated from sample types not integrated in the database in the first place.

This low susceptibility of the method’s efficiency to matrix influences stands in marked contrast to nucleic acid based detection techniques like PCR, which require a very clean starting sample. For cultured organisms PCR works well but has had little success in real-time biodetection of environmental samples owing to inhibition by a myriad of possible interferents. We think that the vibrational spectroscopic approach presented here perfectly compensates for these deficits of PCR methodologies. It displays high robustness concerning matrix interferences, and since reliable results can be obtained within 3 h, point-of-care detection of *B. anthracis* is possible in “real-world samples”.

## Experimental Section

Details of the methods can be found in the Supporting Information. Inactivation of endospores was achieved by exposure to 20 % formaldehyde solution for one hour. A solution of polyvinylpyrrolidone-covered silica colloidal particles diluted in 0.15 m sodium chloride solution was employed as a density gradient medium for the isolation. Endospore enumeration after the isolation step was performed by microbial plating. The Raman spectra were collected with a Raman microspectrometer under ambient conditions on fused-silica substrates with 532 nm excitation. The samples were irradiated with 7 mW with a laser spot of ca. 1 μm during an integration time per endospore of 5 s plus 1 s of preburning. All chemometrical calculations were conducted with Gnu R.

## Supplementary material

Detailed facts of importance to specialist readers are published as ”Supporting Information”. Such documents are peer-reviewed, but not copy-edited or typeset. They are made available as submitted by the authors.
