# Supplementary material for: Raman Spectroscopic Detection of Anthrax Endospores in Powder Samples
Source: Angew Chem Int Ed Engl. 2012 Apr 13;51(22):5339–42. doi: 10.1002/anie.201201266 (PMC3470700; doi:10.1002/anie.201201266)
Supplement: Supplementary file 1 [file anie0051-5339-SD1.pdf]

Angewandte Chemie
Eine Zeitschrift der Gesellschaft Deutscher Chemiker

Supporting Information
© Wiley-VCH 2012
69451 Weinheim, Germany

Raman Spectroscopic Detection of Anthrax Endospores in Powder Samples**

*S. Stöckel, S. Meisel, M. Elschner, P. Rösch, and J. Popp**

anie_201201266_sm_miscellaneous_information.pdf

table of content

Material and Methods 2
  Bacillus strains 2
  Sample preparation 2
  Inactivation and isolation procedures 2
  Spectroscopic instrumentation 3
  Multivariate analysis 3
  Isolation yield determination. 4
Figures 5
Tables 6
References 8

Material and Methods

Bacillus strains

The following strains were examined in this publication: *B. anthracis* 5261 (Federal Research Institute for Animal Health, Jena, Germany), *B. anthracis* Sterne (EQADEBA-Repository, Robert-Koch-Institute, Berlin, Germany), *B. megaterium* DSM 90, *B. mycoides* DSM 299, *B. subtilis* DSM 10, and *B. thuringiensis* DSM 350. All but the *B. anthracis* strains were purchased from the German Collection of Microorganisms and Cell Cultures (DSMZ, Braunschweig, Germany).

Sample preparation

Endospore suspensions with concentrations around 10^7 spores per ml were prepared *via* two different methods: One method was the cultivation on nutrient agar (NA) plates at 30 °C. The medium is formulated as follows: 5.0 g peptone, 3.0 g meat extract, 0.04 g $MnSO_4$ H_2O, 15 g agar and 1000 ml distilled water (pH 7.0 ± 0.2, autoclaved at 121 °C, 20 minutes). The bacteria were rinsed off the plates after seven days of cultivation and washed three times by centrifugation and re-suspension in distilled water. The other cultivation approach at 37 °C relied on yeast extract agar consisting of 10.0 g peptone, 2.0 g yeast extract, 0.04 g $MnSO_4$ H_2O, 15 g agar per 1000 ml aqua bidest (pH 7.0 ± 0.2, autoclaved at 121 °C, 20 min) and was described previously in Stöckel *et al.*[1] Batches of *B. megaterium* DSM 90, *B. mycoides* DSM 299, *B. subtilis* DSM 10, and *B. thuringiensis* DSM 350 were prepared with both procedures, whereas the *B. anthracis* strains were cultivated solely following the second method.
The studied powder matrices were acquired in local common stores. In detail the powders were as follows: baking powder (Back-Gold Backpulver, Meier Pudding, Trittau, Germany), table salt (MarkenSalz, Bad Reichenhaller, Südsalz GmbH, Heilbronn, Germany), gypsum (Sycofix Bau- und Hobby-Gips, Sieder GmbH, Plaue, Germany), baking soda (HausNatron, Lucullus Backen & Genießen GmbH & Co. KG, Darmstadt, Germany), milk powder (Sucofin Magermilchpulver, TSI GmbH & Co. KG, Zeven, Germany), analgesic tablets (Thomapyrin® intensiv, Boehringer Ingelheim Pharma GmbH & Co. KG, Ingelheim am Rhein, Germany), bird sand (Vitakraft Vogelsand, Vitakraft-Werke Wührmann & Sohn GmbH & Co. KG, Bremen, Germany), washing powder (Weißer Riese KraftPulver, Henkel AG & Co. KGaA, Düsseldorf, Germany).
Endospores, coming from at least ten independently cultivated batches for each *Bacillus* species to account for biological variability, were inoculated to the powdery matrices for at least 24 h.

Inactivation and isolation procedures

1.5 ml of 20% formaldehyde solution (Sigma-Aldrich Chemie GmbH, Taufkirchen, Germany) were applied to 100 mg spiked powder to inactivate the potentially pathogenic endospores. The samples were rotated continuously (VWR tube rotator, VWR, Leuven, Belgium) during the time of treatment of one

hour until the process was stopped by centrifugation with 12100g (MiniSpin, Eppendorf, Hamburg, Germany) for one minute.
After three additional washing steps the sediments were suspended into 0.2 ml distilled water. After homogenization via shaking the suspension was carefully layered on top of two density gradient volumes of 0.5 ml (densities of 1.050 g/ml and 1.123 g/ml) in a 1.5 ml micro-tube. The density gradient medium Percoll (Biochrom AG, Easycoll Separating Solution, Berlin, Germany) consisted of PVP (polyvinylpyrrolidone) covered sodium-stabilized silica colloidal particles diluted in 0.15 M sodium chloride solution.[2] The samples were afterwards centrifuged for five minutes at 12100g to form stable density gradient in the vessel. If necessary, like in case of milk powder, light weighted matrix components swam on top of the liquids and were removed. The upper liquid volume was recovered together with the boundary layer to gather most of the less dense endospores. The matrix residue including the denser endospores was afterwards thoroughly mixed again with the remaining supernatant Percoll solution plus 1 ml of added distilled water and was allowed to stand undisturbed for a maximum of five minutes. Most of the present matrix material was soon sedimented so that the supernatant appeared quite clear, which was then abstracted and joined with the first aliquot containing the less denser spores in a 2.0 ml micro-tube. The suspension was finally washed three times with distilled water to get rid of the Percoll residues and stored at 4 °C until further processing.

Spectroscopic instrumentation

All of the Raman spectra were collected under ambient conditions. The Raman spectroscopic measurements were carried out with a micro-Raman device (BioParticleExplorer, rap.ID Particle Systems GmbH, Berlin, Germany) that allows automated measurements of single-cell Raman spectra with an excitation light of 532 nm from a solid-state frequency-doubled Nd:YAG module (LCM-S-111-NNP25, Laser-export Co. Ltd.). An Olympus MPLFLN 100xBD objective focused the Raman excitation light onto the sample with a spot size of <1 µm laterally, so that approximately 7 mW hit the sample. The integration time per Raman spectrum (-113 cm^{-1} – 3186 cm^{-1}) was five seconds after a pre-burning period of one second to mitigate spectral contributions because of fluorescence, though it was already of minor extent in most of the endospore spectra. After removal of the Rayleigh scattering via two edge filters, the 180° back-scattered Raman light was diffracted with a single-stage monochromator (HE 532, Horiba Jobin Yvon) with a 920 lines/mm grating and collected with a thermoelectrically cooled CCD camera (DV401-BV, Andor Technology) with a spectral resolution of ca. 7 cm^{-1}. For single cell measurements, one spectrum of each cell was recorded after 2.5 seconds and one after 5 seconds. These two were afterwards compared for spike removal.

Multivariate analysis

Gnu R was used for the statistical analyses.[3] The procedure mainly consisted of three steps: pre-processing, training of the self-learning machine to build a model and validation. The pre-processing of every dataset was always the same. First the spectra underwent a wavenumber calibration with acetaminophen as standard.[4] Then the background of the spectra and cosmic spikes were removed. The background was stripped off by employing a statistics sensitive nonlinear iterative peak-clipping

algorithm (SNIP), which is in principle a composite of a low statistics filter and a peak clipping algorithm.[5] A forth-order-algorithm was applied with a clipping window of seven. Because of their origin the cosmic spikes are neither correlated in time nor in space and could therefore be localized by recording two Raman spectra of the same endospore. Intensity differences for each channel and their standard deviation were calculated and spikes located in channels, where the intensity difference exceeded twice the standard deviation. After the removal of cosmic spikes a cut off of the fingerprint regions of the spectra took place. For the calculation the wavenumber regions 639 to 1802 cm^{-1} and 2783 to 3186 cm^{-1} were used. A further pre-processing step was normalization: A spectrum was divided by its area, which was calculated as the Euclidean distance of the spectrum to the zero spectrum (2-norm).
To reduce the dimensionality of the problem and to remove white noise a principal component analysis (PCA) was performed.[6] The data were neither scaled by channel nor centered before the PCA. After a particular channel the scores were cut off in the new spectral space. The number of chosen scores correlates with the size of the data set, but a good choice is to use not more than 2-5% of the number of spectra to avoid overfitting.[7] If a new set of spectra had to be labeled the pre-processing of this new dataset was the same as before PCA. To convert both sets in the same spectral space we did not perform a PCA with the combined dataset, but rather rotated the new set by the loadings of the PCA of the first dataset into the spectral space of the first dataset.
We chose a linear discriminant analysis (LDA) as supervised classifier to evaluate the spectral datasets.[8] Cross-validation was used to validate the classifier and this accuracy was taken as accuracy of the classification model. An estimation of the generalization error was done by means of a hold-out technique: Sets of endospores from all the analyzed *Bacillus* species were used for training, other completely independent batches of the same strains, which were separately cultured under exact the same conditions, were used as validation set. In doing so an application of the procedure under realistic conditions was simulated and its accuracy assessed.

Isolation yield determination.

Endospore enumeration after an isolation step was performed by standard microbial plating technique using NA agar plates to assess the recovery performance of the isolation method. Samples of autoclaved baking powder and sand with known inoculation numbers of viable *B. thuringiensis* endospores were prepared trice for each starting concentration, which amounted to 10^8, 10^6, 10^4, and 10^3 cfu/g matrix. All 24 samples were left to stand for 24 h and underwent the isolation procedure afterwards. The resulting suspensions were serially diluted to achieve solutions of appropriate cell concentrations, of which two times 100 µl were put onto NA agar plates. After 24 h of incubation at 30 °C the visible colonies on the plates were enumerated. The two lowest concentrations are obviously too low to pose a threat in, e. g., standard letters but have been considered to approach towards the possible limit of detection. Three replicates of each inoculation concentration and matrix were prepared and subjected to the isolation procedure. Hereby 100 mg of the respected matrix were spiked.

Figures

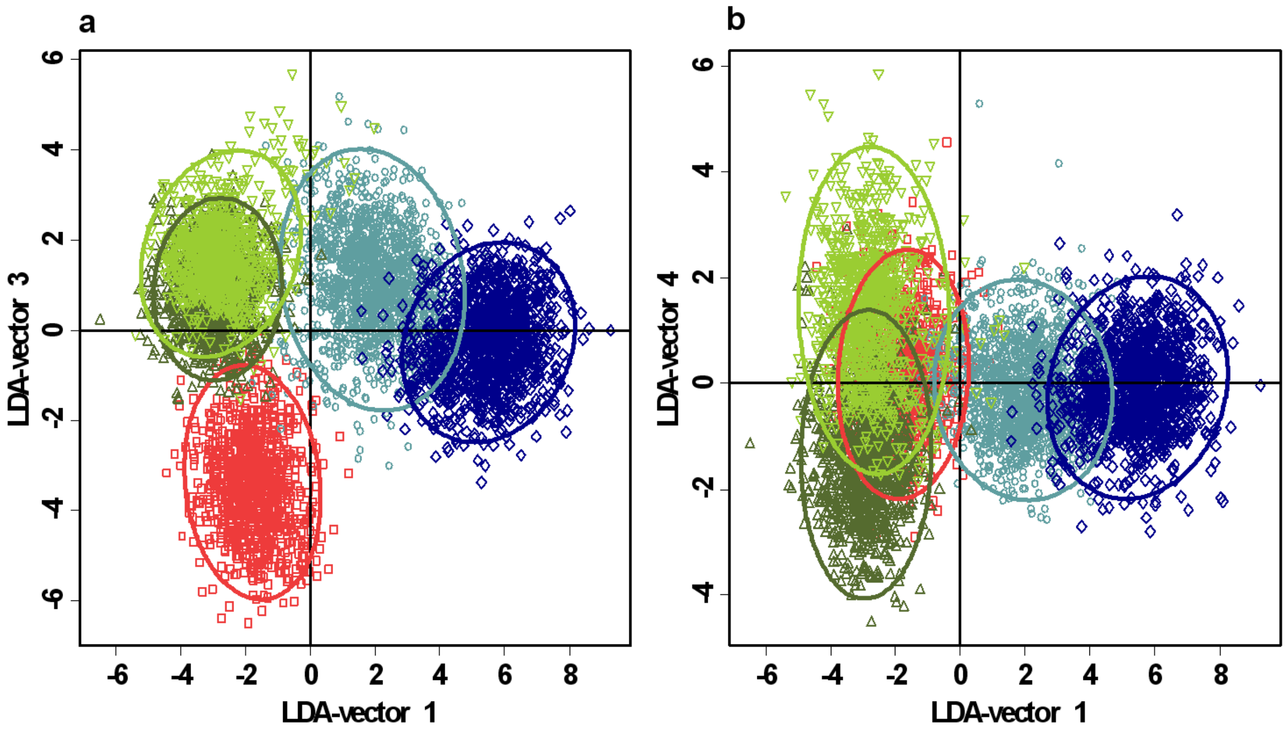

Figure S1. Score plots of the LDA model. a) The spectra are arranged according to their scores of the linear discriminant functions 1 and 3: *B. anthracis* (□), *B. megaterium* (O), *B. mycoides* (Δ), *B. subtilis* (◊), and *B. thuringiensis* (∇). The *B. anthracis* spectra bear negative scores on LD1 and LD3 and therefore cluster in quadrant III. Ellipsoids depict the distribution of the group members via double standard deviation. b) The spectra are arranged according to their scores of the linear discriminant functions 1 and 4: Coding vide supra. Different scores on LD4 of *B. mycoides* and *B. thuringiensis* explain the discrimination between these two classes.

Tables

Table S1. Recovery results for the performed viable cell counting of *B. thuringiensis* endospores. Three baking powder and sand samples á 100 mg were spiked with defined loads of viable *B. thuringiensis* endospores in concentrations from 3.5×10^2 to 3.5×10^7 cfu. The recovered endospores after the isolation were determined twice for each trial via viable cell counting and the average yield per trial is given.

c_0 [cfu/100 mg]	cfu isolated from baking powder			cfu isolated from sand		
	trial 1	trial 2	trial 3	trial 1	trial 2	trial 3
3.5×10^7	2.0×10^5	0.6×10^5	3.3×10^5	3.1×10^5	3.5×10^5	1.8×10^5
3.5×10^5	4.8×10^4	4.8×10^4	6.2×10^4	4.5×10^4	3.1×10^4	2.1×10^4
3.5×10^3	3.7×10^2	5.5×10^2	4.0×10^2	2.1×10^2	3.2×10^2	2.5×10^2
3.5×10^2	1.0×10^1	0.5×10^1	4.3×10^1	1.5×10^1	2.0×10^1	0.7×10^1

Table S2. Summary of the compiled data. a) The number of single endospore Raman spectra per *Bacillus* species measured to build the LDA model is given together with the sample types, from which the endospores had been isolated from (A agar plates, B baking powder, G gypsum, M milk powder, N baking soda (natron), P analgesic tablets (painkiller), S bird sand, W washing powder). Endospores with sample type A were taken directly from agar plates, underwent formaldehyde treatment and were measured without being inoculated into one of the matrices. b) The number of single endospore Raman spectra per *Bacillus* species measured from the validation samples to test the LDA model is given together with the sample types, from which the endospores have been isolated from (C common table salt and rest of abbreviations vide supra).

a)

Bacillus species	no. spectra	isolated from							
B. anthracis 367 & Sterne	997		B					S	
B. megaterium DSM 90	1420	A	B	G	M	N	P	S	W
B. mycoides DSM 299	1142	A	B	G	M	N	P	S	W
B. subtilis DSM 1051	1217	A	B	G	M	N	P	S	W
B. thuringiensis DSM 350	947	A	B	G	M	N		S	W

b)

Bacillus species	no. spectra	isolated from								
B. anthracis 367 & Sterne	242		B					S		
B. megaterium DSM 90	380	A	B			N		S		
B. mycoides DSM 299	402	A	B			N		S		
B. subtilis DSM 1051	331	A	B		M		P		W	
B. thuringiensis DSM 350	295	A	B	G	M					
B. anthracis 367	191									C

Table S3. Results of the cross-validation to evaluate the LDA model classification accuracy. The confusion table depicts the labeling of Raman spectra used to train the LDA classifier after leave-one-out cross-validation including the sensitivities (true positive rates, sens.) and specificities (true negative rate, spec.) per *Bacillus* species. The overall classification rate was 96.5% (5037 spectra of 5218 correctly labeled).

actual / predicted	*B. anthracis*	*B. megaterium*	*B. mycoides*	*B. subtilis*	*B. thuringiensis*	sens. [%]	spec. [%]
B. anthracis	**983**	11	1	0	3	98.6	99.7
B. megaterium	2	**1392**	1	2	3	98.0	99.8
B. mycoides	6	2	**1036**	0	140	90.7	96.7
B. subtilis	0	11	0	**1215**	0	99.8	99.7
B. thuringiensis	6	4	104	0	**801**	84.6	97.6

References

[1] S. Stöckel, W. Schumacher, S. Meisel, M. Elschner, P. Rösch, J. Popp, *Appl. Environ. Microbiol.* **2010**, *76*, 2895-2907.
[2] H. Pertoft, *J. Biochem. Biophys. Methods* **2000**, *44*, 1-30.
[3] R. Development Core Team, R Foundation for Statistical Computing, Vienna, Austria, **2008**.
[4] M. M. Carrabba, in *Handbook of Vibrational Spectroscopy, Vol. 1*, John Wiley & Sons, Ltd, **2006**.
[5] M. Morhác, *Nucl. Instrum. Meth. A* **2009**, *600*, 478-487.
[6] K. Pearson, *Philos. Mag.* **1901**, *2*, 559-572.
[7] V. N. Vapnik, *The nature of statistical learning theory*, 2 ed., Springer, New York, **2000**.
[8] R. Fisher, *Ann. Eugenic.* **1936**, *7*, 179-188.
